# Flow Cytometry Total Cell Counts: A Field Study Assessing Microbiological Water Quality and Growth in Unchlorinated Drinking Water Distribution Systems

**DOI:** 10.1155/2013/595872

**Published:** 2013-06-02

**Authors:** G. Liu, E. J. Van der Mark, J. Q. J. C. Verberk, J. C. Van Dijk

**Affiliations:** ^1^Section Sanitary Engineering, Department of Water Management, Faculty of Civil Engineering and Geosciences, Delft University of Technology, P.O. Box 5048, 2600 GA Delft, The Netherlands; ^2^Dunea Water Company, Plaza of the United Nations 11-15, P.O. Box 756, 2700 AT Zoetermeer, The Netherlands

## Abstract

The objective of this study was to evaluate the application of flow cytometry total cell counts (TCCs) as a parameter to assess microbial growth in drinking water distribution systems and to determine the relationships between different parameters describing the biostability of treated water. A one-year sampling program was carried out in two distribution systems in The Netherlands. Results demonstrated that, in both systems, the biomass differences measured by ATP were not significant. TCC differences were also not significant in treatment plant 1, but decreased slightly in treatment plant 2. TCC values were found to be higher at temperatures above 15°C than at temperatures below 15°C. The correlation study of parameters describing biostability found no relationship among TCC, heterotrophic plate counts, and Aeromonas. Also no relationship was found between TCC and ATP. Some correlation was found between the subgroup of high nucleic acid content bacteria and ATP (*R* ^2^ = 0.63). Overall, the results demonstrated that TCC is a valuable parameter to assess the drinking water biological quality and regrowth; it can directly and sensitively quantify biomass, detect small changes, and can be used to determine the subgroup of active HNA bacteria that are related to ATP.

## 1. Introduction

Microbial regrowth during water distribution is undesirable since the regrowth can lead to hygienic, aesthetic, and technical problems, such as the proliferation of opportunistic pathogenic bacteria [1], an increase in taste and odor [2], and the biocorrosion of pipe material [3]. In practice, there are two approaches to limit the regrowth: maintaining disinfectant residuals and limiting the concentration of growth-promoting compounds in the treated water [4–7].

Biomass quantification is the most direct way to evaluate the microbial regrowth in distributed water. Worldwide, a heterotrophic plate count (HPC) is the primary parameter to quantify biomass [8]. The worldwide accepted guideline for HPC is 20 colony forming units per mL (CFU mL^−1^) for treated water and 300 CFU mL^−1^ for distributed water [9]. In The Netherlands, the regulation for the quality of distributed water has been set to the geometric year mean of 100 CFU mL^−1^. Despite its common use, the cultivation-dependent HPC method is increasingly challenged by the fact that only a small fraction (less than 1%) of the microorganisms in drinking water can be detected [5, 10, 11]. Other limitations, such as the fact that the method is time consuming and the results are influenced by various factors (e.g., temperature and needed culture time), have been well discussed previously [5, 11–14].

In the last decade, cultivation independent detection methods, such as adenosine triphosphate (ATP) [5, 13] and flow cytometry total cell count (FCM-TCC, referred to as TCC afterwards [11, 15, 16]) have gained foremost interest because these methods are accurate, rapid, quantitative, detect both cultivable and uncultivable microorganisms, and are easy to perform. 

ATP is a parameter that assesses active biomass by quantifying the activated, energy-rich metabolic compounds present in all viable cells [17]. ATP has been proven to be a suitable parameter for biomass quantification in drinking water and has found a wide application including the analysis of groundwater [18, 19], assessment of biofilters in water treatment plants [20, 21], drinking water [11, 12, 22], and biofilms in drinking water distribution system (DWDS) [23, 24]. For microbial regrowth in DWDS, research evaluating the influence of water composition, distance, and season on distributed water ATP concentrations [5] has been conducted. As ATP is an indirect biomass quantification method, weak relationships between HPC and ATP were found in all the reported investigations.

Flow cytometry (FCM) is a routine method for characterizing cellular biology that has been used since the 1970s. One promising application of FCM is that it is able to characterize and distinguish different physiological states of microorganisms at the single cell level with high-throughput capacity [25]. As reported by Berney et al. [15], double staining with the nucleic acid-binding SYTO dyes and propidium iodide (PI) was used to distinguish between intact cells and membrane-damaged cells. Although most FCM is not used for the purpose of quantifying microorganisms, it became an attractive method for studying microbial growth in oligotrophic environments, for example, drinking water environments. It has been used for rapid detection bacteria associated with loss of disinfectant residual in drinking water distribution system [26], Hammes et al. [11] developed an FCM quantification method to accurately count microbial cells at concentrations as low as 1,000 cells mL^−1^ and have demonstrated biomass changes during treatment. No relationship between TCC and HPC was observed. However, a good relationship between TCC and ATP was found [11, 12] and an even better relationship between intact biovolume and microbial ATP [13] in drinking water samples was seen. The changes in biomass during drinking water distribution have also been monitored by TCC [4]. However, in this research, only two locations in the distribution system were selected, which were within close proximity of the treatment plant.

The objectives of the present study were (1) to evaluate the application of TCC in DWDS, taking into account the influence of water composition, distance to the treatment plant, and water temperature; (2) to determine the relationship between TCC, ATP, *Aeromonas*, and HPC in distributed drinking water.

## 2. Material and Methods

### 2.1. Selected Treatment Plants and Sampling Locations

In this study, distribution systems of two treatment plants of the Dunea Water Company were selected. Both treatment plants (called, hereafter, TP1 and TP2) take source water from the Meuse River and the water is treated by similar processes. The source water, after pretreatment, is transported over 30 km to a dune area of natural lakes where it is infiltrated by gravity into the subsoil. After an average residence time of 2 months, the water is abstracted from the dunes. Afterwards, water is posttreated by softening, powdered activated carbon, aeration, rapid sand filtration (RSF), and slow sand filtration (SSF) before being pumped into the distribution system. The water is supplied without disinfectant.

A one-year-long monitoring program was carried out in the distribution systems of TP1 and TP2. Samples were taken from the supply area in the surrounding areas of the treatment plants (proximal area), the central part of the distribution systems, and a distal part of distribution systems. The average distance of the proximal, central and distal parts of the distribution system from each production plant is shown in Table 1 and Figure S1 (see supplementary materials available online at http://dx.doi.org/10.1155/2013/595872). To get representative and reliable results, the number of sampling locations was determined based on the supply connections in the area, and samples were taken randomly every month from locations on the same street. In total, 260 samples were collected in 2011. All samples from the distribution systems were taken directly from consumers' taps. Before taking the water samples, each tap was flushed until the water temperature remained stable for 30 seconds (with temperature meter with response time of 10 s, sensitivity of 0.1°C). Water samples were transported within two hours after sampling and stored at 4°C. Analyses were performed within 24 h after water sample collection.

### 2.2. Flow Cytometry Total Cell Count (TCC)

#### 2.2.1. Total Cell Count and Damaged/Intact Cell Count

All collected samples were analyzed by TCC, and damaged cells and intact cells were distinguished by flow cytometer measurements. Staining and flow cytometry were done as described previously [11, 15]. In short, for working solutions, SYBR Green I (SG) (Invitrogen AG, Basel, Switzerland) was diluted 100× in anhydrous dimethylsulfoxide (DMSO) and propidium iodide (PI; 30 mM) and was mixed with the SG working solution at a ratio of 1 : 50 (SGPI). Both working solutions were stored at −20°C until use. 

From every water sample analysis, two 1 mL subsamples were prepared and stained with both SG (for total cell counting) and SGPI (for intact cell counting) at 10 *μ*L mL^−1^. Before analysis, samples were incubated in darkness for 10 min (SG) and 15 min (SGPI). Prior to flow cytometric analysis, the water samples were diluted with 0.22 *μ*m filtered, commercially available bottled water to 10% v/v of the initial concentration. Recalculation of the actual cell number was made based on the dilution ratio. TCC was performed with a BD ACCURI C6 flow cytometer (United States). Green fluorescence was collected at 520 ± 10 nm, red fluorescence above 630 nm, and high-angle sideward scatter (SSC) at 488 nm. 

#### 2.2.2. Low Nucleic Acid Content (LNA) Bacteria and High Nucleic Acid Content Bacteria

**Figure 1:**
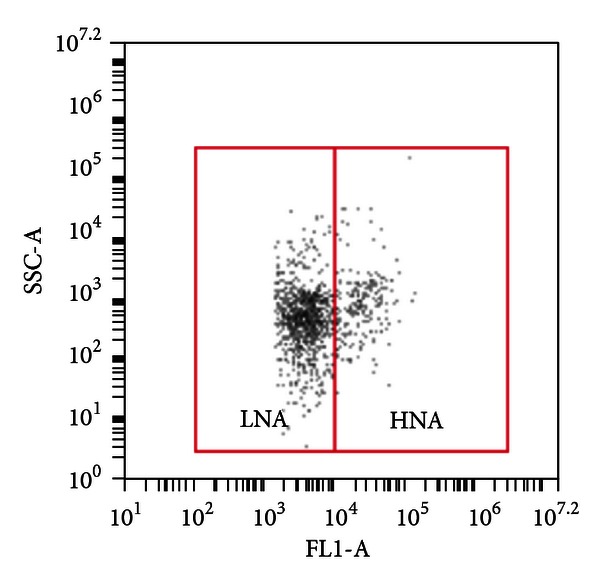
FCM results are represented in dot-plots of total cell counts, and low nucleic acid content (LNA) bacteria and high nucleic content acid (HNA) bacteria are indicated by two solid lines. LNA and HNA are two groups of bacteria generally classified by FCM measurements. Complete and original plot with noise background is shown in Figure S2.

The fluorescence intensity measured by FCM (indicated as FL1-A in Figure 1) has been used as an indicator of apparent cellular nucleic acid content [28, 29] and sideward scatter (SSC) has been applied as an indication of cellular size [28–30]. Based on the distinctly different fluorescence intensity and SSC signals detected by FCM in combination with nucleic acid stains, bacteria have been broadly classified into two groups: low nucleic acid content (LNA) bacteria and high nucleic acid content (HNA) bacteria. LNA bacteria and HNA bacteria were distinguished and quantified according to two clusters in FCM dot-plots of FL1-A and SSC-A (Figure 1 and Figure S2).

### 2.3. Adenosine Triphosphate (ATP)

All collected samples were analyzed for ATP. Total ATP concentration was determined as described previously using the BacTiter-Glo reagent and a luminometer [20]. In short, ATP was released from suspended cells with nucleotide-releasing buffer (NRB, Celsis), and the luminometer was calibrated with solutions of free ATP (Celsis) in autoclaved tap water following the procedure as given by the manufacturer. The detection limit was 1 ng ATP L^−1^.

### 2.4. Heterotrophic Plate Count (HPC)

Water samples were analyzed on HPC. The water samples were diluted tenfold in sterile drinking water and 50 *μ*L of the appropriate dilution was spread in triplicate over the surface of R2A agar plates (Oxoid Led). Subsequently, agar plates were incubated at 25°C for 10 days. The number of colony forming units (CFUs) was determined.

### 2.5. Aeromonas

Samples were analyzed by *Aeromonas* plate counts. After filtering 100 mL water samples through a 0.45 *μ*m filter, the filter was incubated with *Aeromonas* dextrin agar, and agar plates were incubated for 24 h at 30°C before the number of CFUs was determined.

### 2.6. Statistical Analyses

The obtained data were statistically analyzed to determine whether TCC and ATP values were significantly different. The normally distributed data were tested by the *t*-test. The nonnormal distributed data were tested by nonparametric tests (Kruskal-Wallis tests). Differences were considered significant when the *P* value was lower than 0.05. Correlation analyses were done by determining Pearson's (to test for linear correlations) and Spearman's rho (to test for nonlinear correlations) correlation coefficients between paired values of TCC and ATP, TCC, and HPC. All statistical calculations were performed with Minitab 16.0.

## 3. Results and Discussion

### 3.1. Microbiological Quality of the Water at the Two Treatment Plants

Table 1 includes the results of the microbiological parameters at TP1 and TP2. From these data it can be concluded that both distribution systems were supplied by biologically stable water.

**Figure 2:**
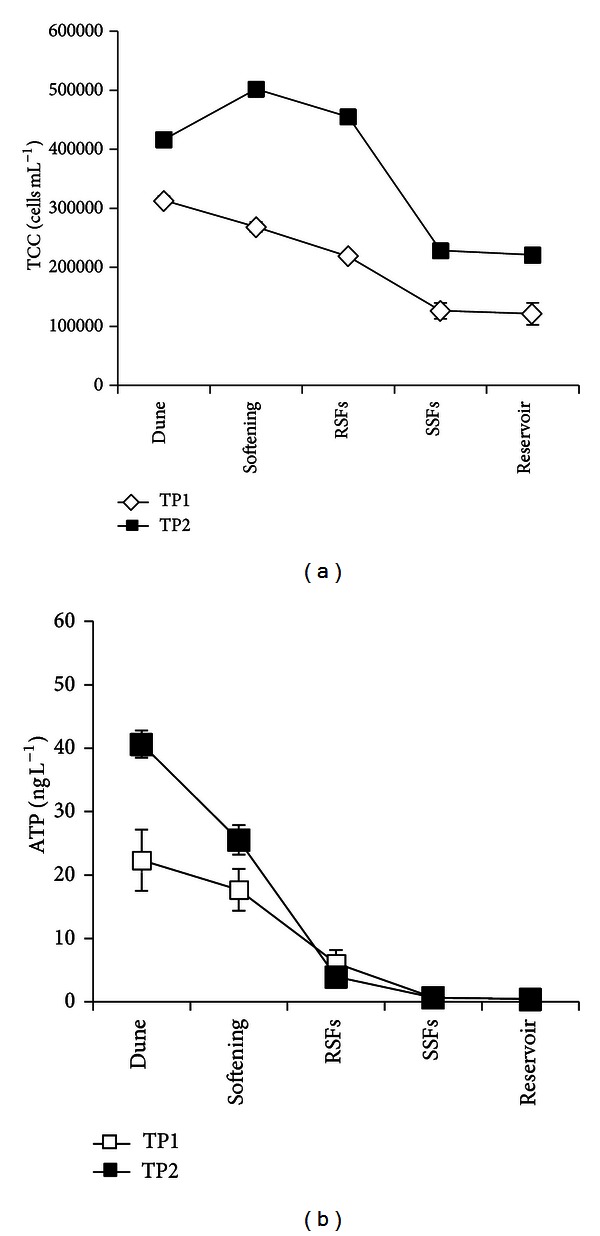
Changes of biomass during drinking water treatment: total cell concentration (a) (*n* = 3) and ATP concentration (b) (*n* = 3). RSF = rapid sand filtration; SSF = slow sand filtration.

Figure 2 gives the changes in the ATP and TCC numbers in the treatment train. It can be concluded that biomass was efficiently removed by the multiple barriers treatments: TP1 removed 62% TCC and 98% ATP; TP2 removed 47% TCC and 99% ATP. Comparing results from TP1 and TP2, the values in abstracted water from dune area of TP2 were higher than that of TP1. Since the same pretreated water is transported to the dune areas, the different values in abstracted water are probably related to the different soil compositions in the two dune areas. Another notable observation is that although ATP and TCC concentrations were generally decreasing in every treatment unit, an increase in TCC after softening in TP2 was observed. This may be related to some biological growth in TP2, possibly caused by the softening chemical. Moreover, the long-term monitoring of treated water AOC content in the two treatment plants (Figure S3 in supplementary data) revealed that TP1 has a stable performance, while some fluctuation was observed in TP2. However, these aspects were not within the scope of the current study. Detailed studies are necessary to understand the differences in performance of the two dune areas and the two treatment plants.

### 3.2. TCC in Distributed Drinking Water

#### 3.2.1. Effect of Water Composition on TCC Levels in Distributed Drinking Water

**Figure 3:**
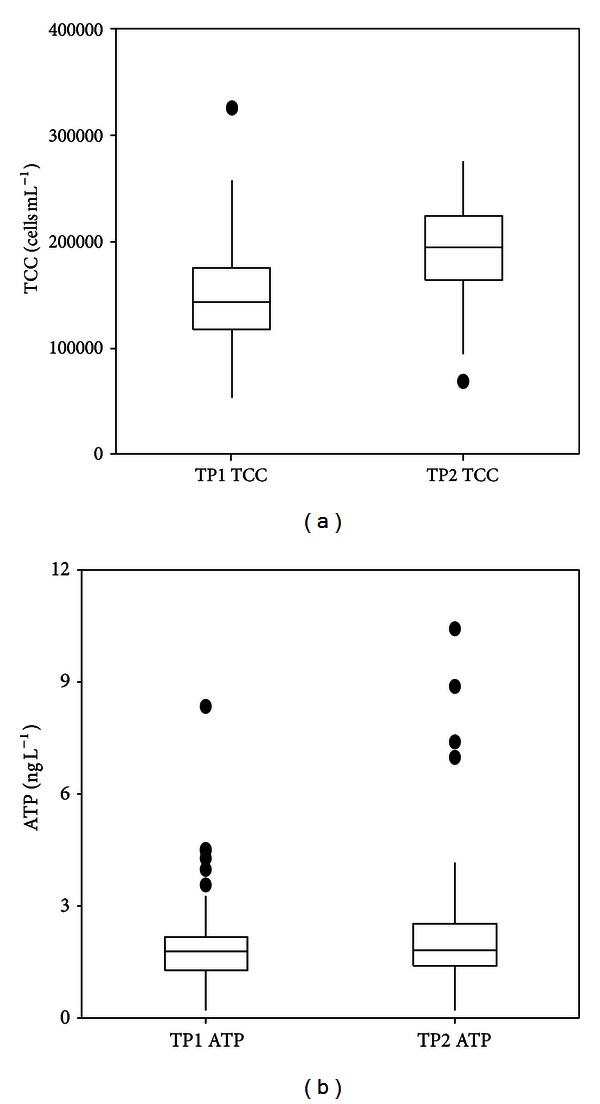
Boxplot of overall TCC (a) and ATP (b) results from distribution systems of TP1 (*n* = 150) and TP2 (*n* = 110).

The TCC in the unchlorinated, distributed drinking water sampled from both distribution systems ranged from 0.5 × 10^5^ cells mL^−1^ to 3.2 × 10^5^ cells mL^−1^ (Figure 3(a), shown as boxplot). According to the literature, these values are typical for the cell concentration of unchlorinated drinking water [4, 11]. The damaged cells and intact cells were distinguished by flow cytometer measurements. It was found that most of the cells (more than 80%) were alive/intact (Figure S4 in supplementary data). That is related to the fact that no disinfectant is being used. TCC values in the TP2 system were significantly higher than those of the TP1 system (*P* < 0.05). This difference in TCC values originated at the treatment plants; it is consistent with the treated water AOC values (Table 1). The treated water of TP1 had relatively lower AOC than TP2. So it may be concluded that the water in the supply area of TP1 is slightly more biologically stable than the water from TP2. However, it should be noted that both TCC and AOC values of both systems are very low compared to studies in the literature.

The ATP concentrations from the two systems did not show significant differences (Figure 3(b)). ATP concentrations were almost all below 3 ng L^−1^ with few outliers. So, it may be concluded that the TCC analysis was better suited than the ATP method to pick up the slight changes in biomass that occurred in the distribution area of TP2. 

#### 3.2.2. Effect of Distance to Treatment Plant on TCC Levels in Distributed Drinking Water

**Figure 4:**
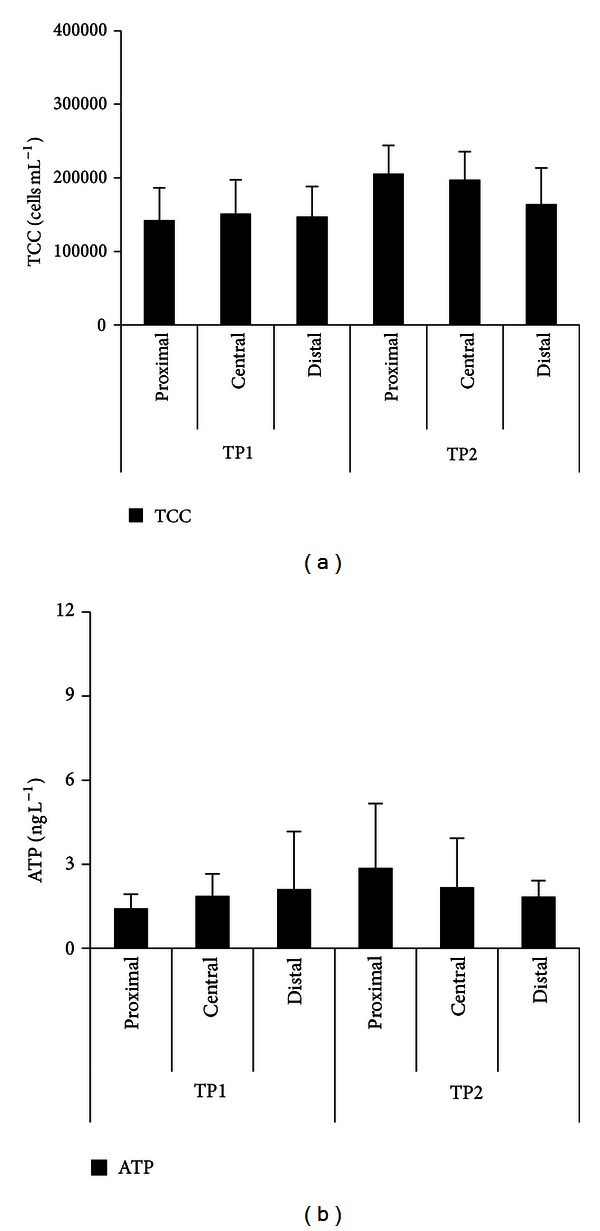
Average TCC (a) and ATP (b) concentrations (with standard deviations) in drinking water sampled from different areas in distribution systems of TP1 and TP2.

The differences in TCC values in the distributed water sampled from proximal, central and distal parts of the distribution systems are shown in Figure 4(a). The TCC values in distributed water did not change significantly during transportation to the distal part of the TP1 system. For the TP2 system, a significant decrease in TCC was observed (*P* < 0.05; Figure 4(a)). For both of the systems, the changes measured by ATP between proximal and central distribution areas were not significant (Figure 4(b)). These results confirm that the water quality of TP1 is slightly better than at TP2, even though both systems can be characterized as biologically stable. Stable trends of biological water quality in unchlorinated distribution systems have been reported by a TCC study of two locations within 6 km from a treatment plant (i.e., Zurich, Switzerland: treated water AOC 32 *μ*g L^−1^ [4]) and by an ATP study in several distribution systems (i.e., the Netherlands: treated water AOC 1.9–14.2 *μ*g L^−1^ [5]). It is important to note that both aforementioned studies concluded that the substrate (AOC, dissolved organic carbon, and/or biodegradable organic matter) decreases corresponding to bioregrowth in distribution systems. Hammes et al. [4] found that a TCC increase corresponded to a decrease in AOC and dissolved organic carbon (DOC) during water treatment, while stable TCC values were found in distributed water where DOC and AOC changed minutely during distribution. In the last study, ATP concentrations decreased in a larger distribution system (the distal area averaged 40 km from the treatment plant, that is, more than two times farther than the two systems in the present study) when the distributed water reached the distal distribution area. The decrease has been attributed to lower biodegradable organic matter concentrations in the distal part of the distribution system due to biological processes and sedimentation during water distribution. In the present study, monitoring of straightforward parameters for substrate (DOC; Figure S5 and Figure S6, supplementary information) and biomass (TCC and ATP) showed only slight changes, suggesting that the treated water of the two selected treatment plants had a high level of biological stability.

The processes occurring during drinking water distribution, such as biofilm formation and detachment, loose deposits accumulation and resuspension, all have an impact on biomass concentration in the bulk water and are strongly related to hydraulic conditions. It is important to mention that the lower biomass concentration measured in distributed water by TCC (in the present study) and ATP (in [5]) in the distal supply area can be caused by biomass enrichment in DWDS either by cells attached as biofilm on the pipe wall surface [31] or by cells associated with particulate matter such as suspended solids and loose deposits [31, 32]. Detailed studies on biomass in different phases in DWDS, such as pipe wall biofilm and loose deposits, are necessary before such a conclusion can be made.

#### 3.2.3. Effect of Temperature on TCC Levels in Distributed Drinking Water

Higher water temperatures promote bacterial regrowth during distribution [33–35]. Over the study period, the water temperature ranged from 4.5°C to 19.5°C. It was observed that water samples could be collected with lower temperatures in the summer time and with higher temperatures in the spring. Therefore, the results in this study are being presented based on different temperatures instead of using general seasonal differences. An earlier statistical study of coliform occurrences in 31 water systems in North America [35] found significant differences in the occurrence of coliform bacteria between water samples with temperatures lower than 15°C and those with temperatures higher than 15°C.

**Figure 5:**
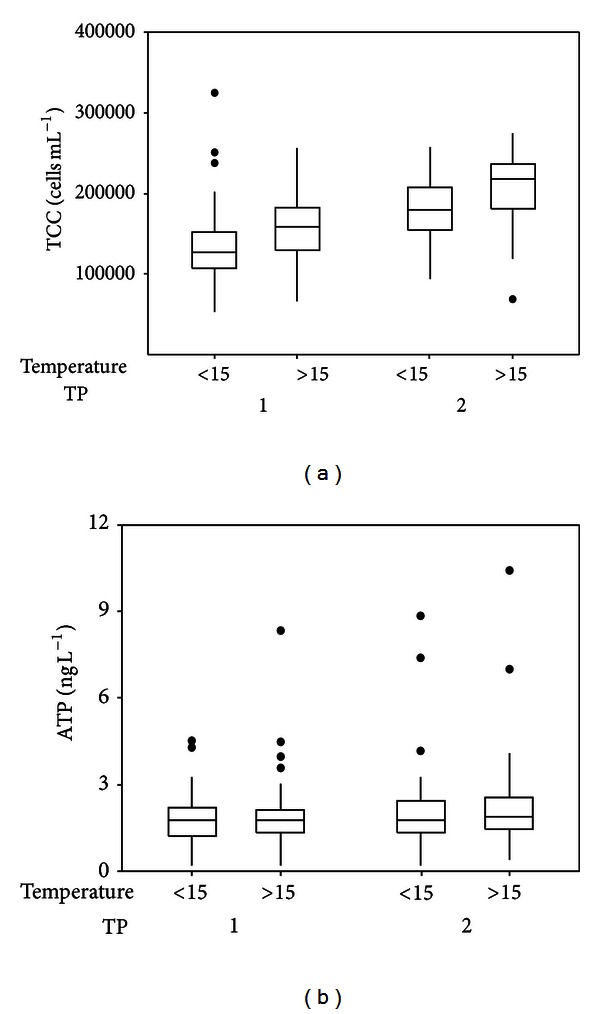
Boxplot of TCC (a) and ATP (b) concentrations in distributed drinking water sampled from two systems at temperatures below 15°C (<15) and above 15°C (>15).

In the present study, the same boundary temperature (15°C) was used to compare the influence of temperature on biomass in the distributed drinking water (Figure 5). The average TCC values at temperatures higher than 15°C were significantly higher than the values of samples collected with temperatures lower than 15°C (Figure 5(a): *P* < 0.05). Again, the same influence was not found in the ATP results, corroborating the higher sensitivity of the TCC measurements (Figure 5(b)). In a recent investigation, no correlation was found between increased temperatures and increased cell concentrations [4], but this may be caused by the limited number of sampling points (only two locations) and the limited distance from the feed reservoir (1 km and 6.1 km).

In another investigation carried out in The Netherlands, low temperature was found to limit microbial activity as measured by ATP [5]. The different results found in this current study can be explained by the greater biological stability of the treated water. In The Netherlands, AOC and BFR tests are used together to evaluate biological stability. BFR is used as a measure for the regrowth potential of drinking water [27]. In DWDS, high BFR was found to be consistent with high concentration of ATP in distributed water [5]. With similar AOC concentrations, BFR values in the systems selected in the present study were 25–47 times lower than those of the systems in the previous study done by van der Wielen and van der Kooij [5]. 

### 3.3. Relationship between TCC and ATP, HPC, and *Aeromonas* in Distributed Drinking Water

**Figure 6:**
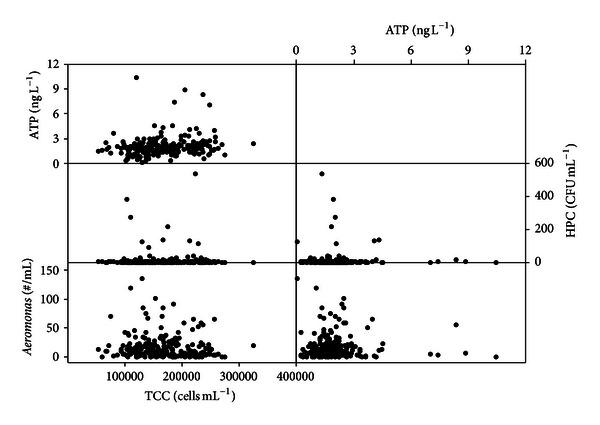
Matrix diagram of TCC, ATP, and *Aeromonas*, HPC, and ATP (*n* = 260).

All of the samples (*n* = 260) were analyzed using ATP, HPC, *Aeromonas,* and TCC. The observed HPC values were within the water quality regulations. The correlation analysis between TCC, ATP, HPC, and *Aeromonas* demonstrated that none of the parameters had a good correlation with each other (Figure 6). Although the TCC is a powerful technique, the weak correlation with *Aeromonas* indicated that it cannot replace *Aeromonas* measurements in the regular biological water quality monitoring. The weak relationship between TCC and HPC has been reported before [4, 5, 11, 12]. These weak relationships are logical, as HPC counts only cultivable cells and *Aeromonas* is only a limited group of the cultivable cells, whereas TCC counts all cells.

In the literature a good relation between TCC and ATP has been reported, in which TCC was measured either by FCM [12, 13] or by microscopy-based cell counts [5]. However, a good correlation between TCC and ATP was not found in the current study (Figure 6). With TCC ranging from 0.5–3.2 × 10^5^ cells mL^−1^, ATP concentrations were found to be relatively stable, mainly between 1–3 ng L^−1^ with a few outliers (Figure 3(b)). By comparing data reported in the literature [12, 13] in which strong correlations were found against the results obtained from the current study, it can be seen that only a limited number of the analyzed samples in the reported research had an ATP concentration lower than 3 ng L^−1^ (0.06 nM). For example, less than 8% of the samples in the study on different aquatic samples (*n* = 102, *R* ^2^ = 0.8; [13] and less than 10% of the samples in the study on distributed water (*n* = 200, *R* ^2^ = 0.69; [12] had ATP concentrations lower than 3 ng L^−1^. As Figure 8 shows, the current research revealed a correlation at ATP concentrations above 3 ng L^−1^. It is possible that the correlation between TCC and ATP occurs only at ATP concentrations above 3 ng L^−1^. 

### 3.4. Relationship between HNA Bacteria and ATP

**Figure 7:**
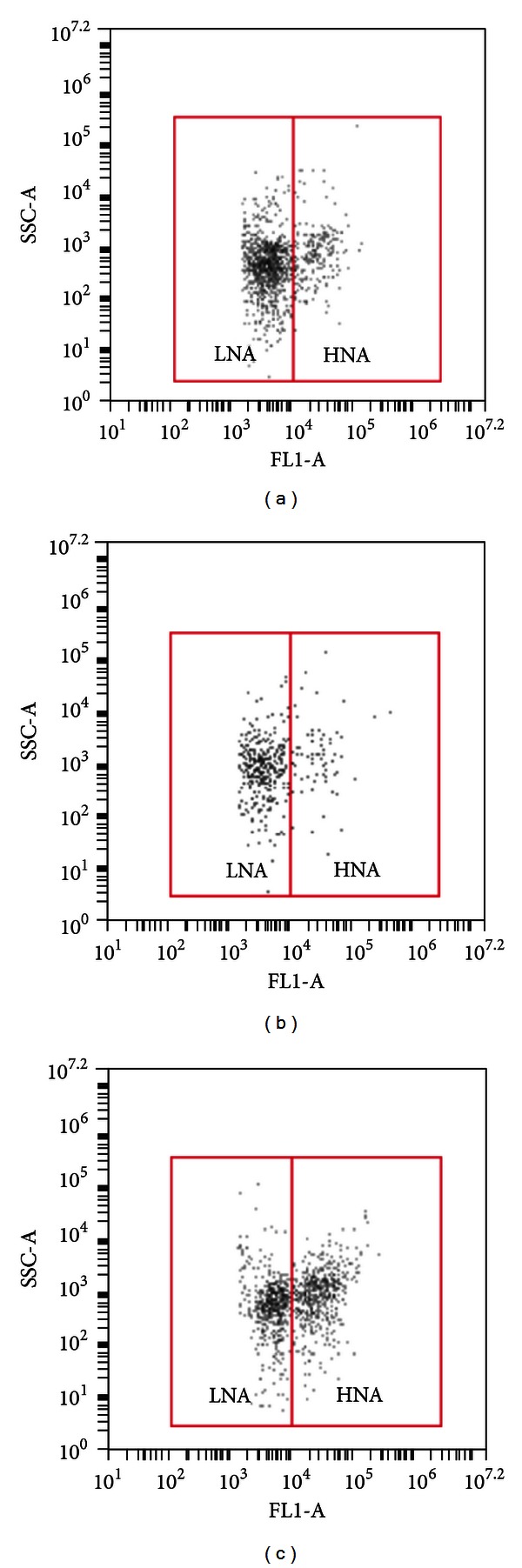
FCM dot-plots of total cell counts of selected FCM measurements based on ATP values. LNA and HNA are indicated by two solid lines. (a) 90,000 cells mL^−1^, 2.0 ng ATP L^−1^; (b) 210,000 cells mL^−1^, 2.0 ng ATP L^−1^; (c) 220,000 cells mL^−1^, 8.4 ng ATP L^−1^.

**Figure 8:**
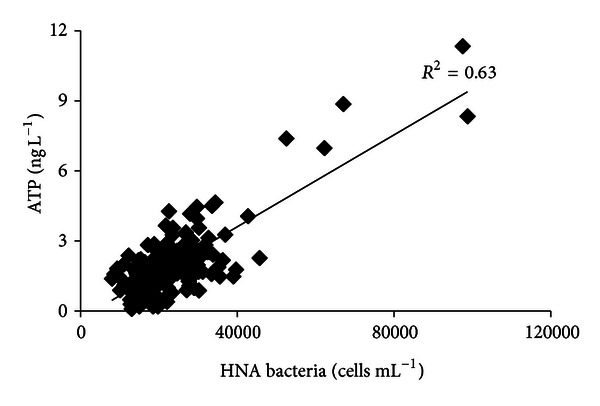
Correlation between HNA bacteria and ATP concentrations driven from the overall data set (*n* = 260).

Theoretically, a correlation between the number of active bacteria and the ATP concentrations can be expected. It should be noticed that the intact LNA bacteria have been considered inactive [36], therefore, may have no contribution of ATP concentration. Therefore, the correlation between cell counts of HNA bacteria and ATP concentrations was studied based on dot-plots of LNA and HNA bacteria from FCM measurements (Figure 7). As shown in Figures 7(a) and 7(b), the two samples contained similar ATP concentrations, but the sample shown in Figure 7(b) had a much higher TCC than the sample shown in Figure 7(a). It is interesting to note that most of the cells in Figure 7(b) were classified as LNA bacteria. Similarly, Figures 7(b) and 7(c) show two samples with similar TCC values, while the increase of HNA bacteria was related to an increase of ATP from 2.0 ng L^−1^ to 8.4 ng^−1^. It is, therefore, hypothesized that ATP concentrations correspond better to the number of HNA bacteria. To confirm this hypothesis, ATP was correlated to the number of HNA bacteria in all of the 260 samples. Indeed, a better correlation was found between HNA bacteria and ATP (Figure 8, *R* ^2^ = 0.63) than the correlation between TCC and ATP (Figure 6, *R* ^2^ = 0.002). The correlation was not as good as reported in previous research [12, 13]. Again, this may be because the ATP concentration was mainly lower than 3 ng L^−1^. 

## 4. Conclusions

(i) The TCC levels were 1.2 × 10^5^ cells mL^−1^ and 2.2 × 10^5^ cells mL^−1^ for treated water at TP1 and TP2, respectively. Based on the levels of TCC, ATP, AOC, and BFR, it is concluded that both TP1 and TP2 produce biologically stable water. Nevertheless, TCC and AOC results demonstrated that the biological quality of treated water at TP1 is slightly better than that at TP2. The differences may result from different soils in their respective dune areas and differences in the treatments. Further investigation is needed to understand these differences.
(ii) TCC values in unchlorinated distributed water averaged between 0.5 and 3.2 × 10^5^ cells mL^−1^. In both distribution systems, the water remains biologically stable and there is no growth, as indicated by the stable levels of TCC and ATP. As revealed by TCC measurements, the biological quality of the distributed water at TP1 is slightly more stable than at TP2. TCC values were found to be higher at temperatures higher than 15°C than at temperatures lower than 15°C.
(iii) Significant changes in TCC values were observed in water with different compositions, different supply areas, and different temperatures. The changes measured by ATP were not significant. ATP and TCC analysis demonstrated that when the ATP concentrations in distributed drinking water are too low to observe significant changes, TCC is a more sensitive method to monitor the biomass changes.
(iv) There is no direct relationship between TCC and ATP, HPC, or *Aeromonas* numbers in distributed drinking water characterized as biologically stable with low ATP concentrations. It was found that in unchlorinated distributed drinking water, metabolic activity (as measured by ATP concentrations) showed a better relationship with the number of HNA bacteria. The extra information on subgroups of LNA and HNA is a clear advantage of TCC over HPC and ATP for bioregrowth study.
(v) Based on this investigation, it is concluded that it is useful to include TCC by FCM on a regular basis to assess the biological quality and stability of distributed drinking water. The TCC quantifies the total biomass and can detect small changes directly and sensitively. Moreover, it can determine the subgroup of HNA, which are related to the active cells and the ATP concentration.

## Supplementary Material

Six figures in four pages of supplementary material have been included in this file for the paper “Flow cytometry total cell counts: A field study Assessing Microbiological Water Quality and Growth in Unchlorinated Drinking Water Distribution Systems”. These Figures presents the description of sampling area, original dot plots generated by flow cytometer, AOC and DOC data from the treatment plants and distribution areas, and the viability of cells detected by flow cytometer.

## Figures and Tables

**Table 1:** The source water and biological quality of treated water of selected treatment plants.

Parameters	TP1	TP2
Source water	AAR water	AAR water
AOC (*μ*g C L^−1^)	5 (±2)	11 (±8)
BFR (pg ATP cm^−2^ day^−1^)	0.73	0.61
TCC (cells mL^−1^)	120000 (±25000)	220000 (±30000)
ATP (ng L^−1^)	0.5	0.5
Studying period	Jan. 2011–Dec. 2011	
Distance (km)		
Proximal	5 ± 0.8	4 ± 0.8
Central	11 ± 1.5	9 ± 1.1
Distal	15 ± 3	16 ± 2

*In the Table: AAR stands for artificial aquifer recharge; AOC (assimilable organic carbon), was averaged values measured from 2006 to 2012 and measured as described by van der Kooij [22]. For both treatment plants, *N* = 25. Detailed results are shown in supplementary data, Figure *S*3. BFR (biofilm formation rate), was measured in 2009 according to methods described by Van der Kooij [27]. ATP and TCC were measured monthly at treatment plants during this study.
