# Supplementary material for: Flow Cytometry Total Cell Counts: A Field Study Assessing Microbiological Water Quality and Growth in Unchlorinated Drinking Water Distribution Systems
Source: Biomed Res Int. 2013 Jun 2;2013:595872. doi: 10.1155/2013/595872 (PMC3684093; doi:10.1155/2013/595872)
Supplement: Supplementary file 1 — Six figures in four pages of supplementary material have been included in this file for the paper “Flow cytometry total cell counts: A field study Assessing Microbiological Water Quality and Growth in Unchlorinated Drinking Water Distribution Systems”. These Figures presents the description of sampling area, original dot plots generated by flow cytometer, AOC and DOC data from the treatment plants and distribution areas, and the viability of cells detected by flow cytometer. [file 595872.f1.docx]

**Supplementary information for field study of flow cytometry cell counts in drinking water distribution system**


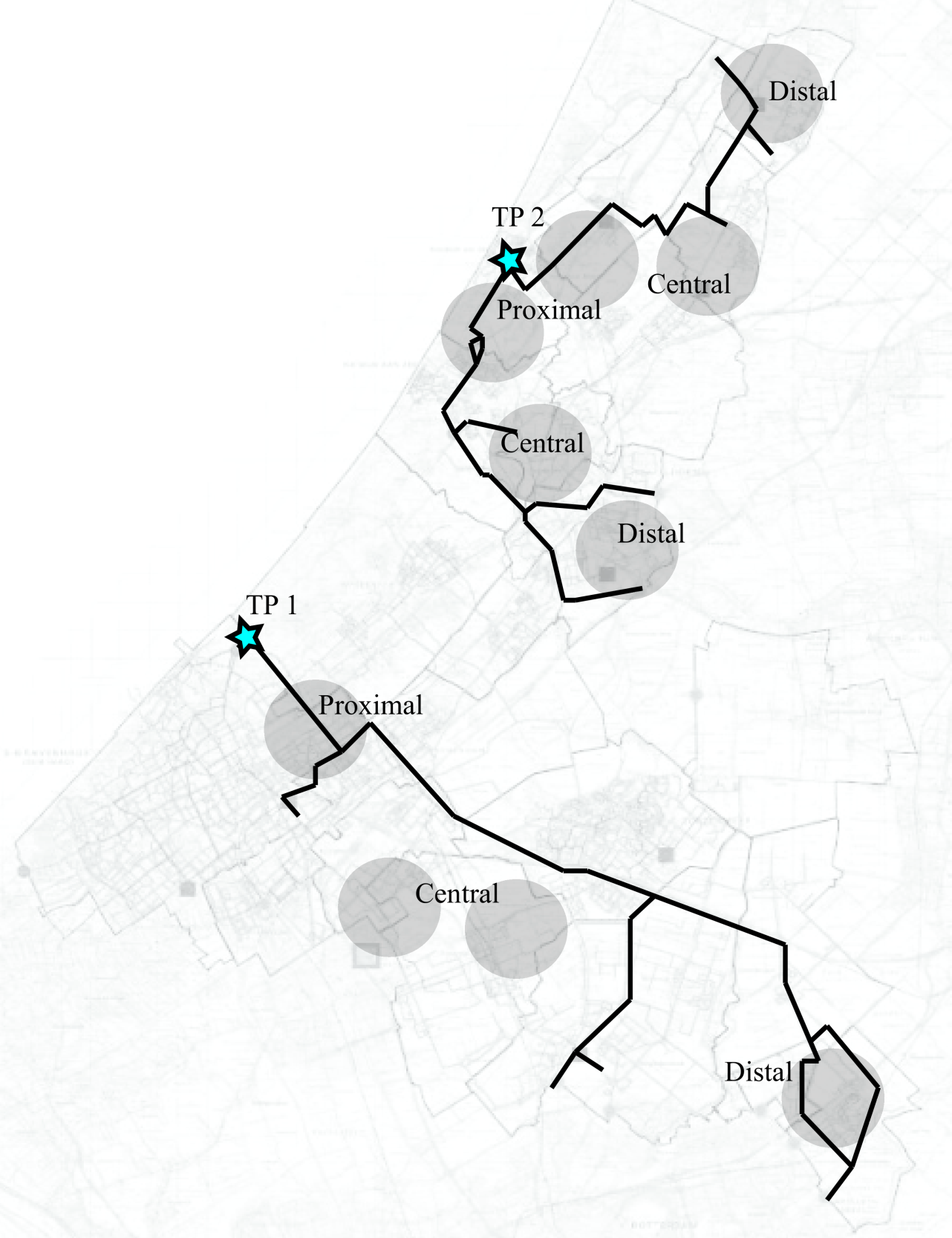


*Figure S1. Schematic diagram of sampling areas in the two distribution systems*





*Figure S2. Original dot plots generated by flow cytometer, with background noise. Gate P1 indicates the noise for LNA bacteria, gate P3 indicates the plot area for cell counting. Gate P1 and P3 were obtained by experience with experience on dealing with huge data base of drinking water samples.*

*Figure S3. historical AOC values of the two treatment plants*

*Figure S4. percentage of intact cell of distributed water from different areas*

*Figure S5. Boxplot of DOC results from distribution system of two treatment plants (TP1, n=34; TP2, n=49)*

*Figure S6. Average DOC concentrations (with standard deviations) in drinking water sampled from different areas in distribution systems of TP1 and TP2 (TP1, n=34; TP2, n=49)*
